# Author response to: Comment on: Effect of centre volume on pathological outcomes and postoperative complications after surgery for colorectal cancer: results of a multicentre national study

**DOI:** 10.1093/bjs/znae193

**Published:** 2024-07-24

**Authors:** Matteo Rottoli, Antonino Spinelli, Gianluca Pellino, Alice Gori, Giacomo Calini, Maria E Flacco, Lamberto Manzoli, Gilberto Poggioli

**Affiliations:** Surgery of the Alimentary Tract, IRCCS Azienda Ospedaliero-Universitaria di Bologna, Bologna, Italy; Department of Medical and Surgical Sciences, Alma Mater Studiorum University of Bologna, Bologna, Italy; Department of Biomedical Sciences, Humanitas University, Milan, Italy; Colorectal Surgery Unit, IRCCS Humanitas Research Hospital, Milan, Italy; Department of Advanced Medical and Surgical Sciences, Università degli Studi della Campania Luigi Vanvitelli, Naples, Italy; Colorectal Surgery, University Hospital Vall d’Hebron, Barcelona, Spain; Surgery of the Alimentary Tract, IRCCS Azienda Ospedaliero-Universitaria di Bologna, Bologna, Italy; Department of Medical and Surgical Sciences, Alma Mater Studiorum University of Bologna, Bologna, Italy; Surgery of the Alimentary Tract, IRCCS Azienda Ospedaliero-Universitaria di Bologna, Bologna, Italy; Department of Medical and Surgical Sciences, Alma Mater Studiorum University of Bologna, Bologna, Italy; Department of Environmental and Preventive Sciences, University of Ferrara, Ferrara, Italy; Department of Medical and Surgical Sciences, Alma Mater Studiorum University of Bologna, Bologna, Italy; Surgery of the Alimentary Tract, IRCCS Azienda Ospedaliero-Universitaria di Bologna, Bologna, Italy; Department of Medical and Surgical Sciences, Alma Mater Studiorum University of Bologna, Bologna, Italy

*Dear Editor*

In response to our article^1^, Joyce *et al*.^2^ raise a question of paramount importance—should all colorectal cancer surgery be centralized? While it is true that some of the outcomes (number of lymph nodes and rates of mortality, severe complications, and neoadjuvant therapy in rectal cancer) are significantly improved in high-volume centres, others (radicality of resection) do not follow the same linear association, at least in our series. This could be explained by the higher rate of very low rectal resections, which are more likely to be performed in high-volume centres.

Although it could be reasonably expected that postoperative and oncological outcomes could improve in high-volume centres, the social cost of a blind centralization of all cases may be worse than its advantages. In our opinion, appropriate oncological care of patients with colon cancer should also be guaranteed in rural areas and isolated territories, with no need for patients to travel far from home. Moreover, colon cancer is more likely to present as an emergency, the treatment of which requires a high level of surgical skill.

Conversely, rectal cancer care requires a level of expertise that is more strongly volume related, as well as hospital services (interventional radiology, surgical endoscopy, pathology, and oncology) that are essential for the treatment of postoperative complications. For these reasons, rectal cancer surgery should only be performed in high-volume centres, based on a threshold that must be accurately assessed in the different national settings.
